# The Adsorption Layer in the System: Carboxymethylcellulose/Surfactants/NaCl/MnO_2_

**DOI:** 10.1007/s11743-012-1340-5

**Published:** 2012-03-14

**Authors:** Elżbieta Grządka

**Affiliations:** Department of Radiochemistry and Chemistry of Colloids, Faculty of Chemistry, Maria Curie-Skłodowska University, M. Skłodowskiej, Curie 3 Sq., 20-031 Lublin, Poland

**Keywords:** Polysaccharide, Surfactant, Adsorption, Zeta potential, Adsorption layer thickness

## Abstract

The influence of surfactants (anionic sodium dodecyl sulfate, and nonionic *tert*-octylphenol ethoxylate with 9.5EO) and their mixtures on the adsorption of carboxymethylcellulose (CMC) in the presence of 0.001 M NaCl on the manganese dioxide surface (MnO_2_) was studied. The increase in CMC adsorption was observed in all measured systems in the presence of surfactants. The reason for this is the formation of complexes between polymer macromolecules and surfactants. Moreover, the dependence between the amount of surfactants adsorption and the CMC initial concentration was also studied. It proves that surfactant adsorption does not depend on the initial concentration of CMC. Another observation is that the increase in pH caused the decrease in CMC adsorption. The explanation of this phenomenon is connected with the influence of pH on the dissociation degree of the polyelectrolyte, kind and concentration of the surface active groups of the adsorbent. To characterize the compact and diffuse adsorption layer the surface charge density and the zeta potential of MnO_2_ in the presence of CMC and surfactants were measured. The surface charge density of MnO_2_ decreases in the presence of CMC or CMC/surfactant complexes. This is due to the presence of negatively charged groups in the compact part of the electric double layer. The zeta potential of MnO_2_ is also lower in the presence of CMC and the CMC/surfactants complexes. The main reason for that is the shift of the slipping plane towards the bulk solution.

## Introduction

The adsorption of polysaccharides on the solid is a very sophisticated process determined by many factors. These factors can be divided into three categories. The first one, connected with the polysaccharide, includes such parameters as molecular weight of the polymer used, its polydispersity, purity and chemical character. The second, related with the adsorbent, includes its chemical character, surface charge, specific surface area, homogeneity and purity. The last category depends on the solution, where the most important parameters are the type of background electrolyte, its ionic strength, pH of the solution and the presence of other substances in the solution such as surfactants. Because of so many parameters which have an influence on the adsorption of polysaccharides, the mechanism of this process has not been fully explained. The adsorption of polysaccharides on the surface of solids has been widely studied with hydrogen bonding and hydrophobic interaction as the primary adsorption mechanisms [1, 2]. However, according to other scientists [3–5], the adsorption of polysaccharides results from an acid–base reaction between the polysaccharide active groups and the metal hydroxyl groups from the solid.

Carboxymethyl cellulose (CMC) is a polysaccharide widely used in the adsorption processes. This abbreviation might be confusing because it is also used for critical micelle concentration. To avoid misunderstanding in this paper the abbreviation ‘CMC’ is used for carboxymethyl cellulose, whereas the abbreviation ‘c.m.c’ stands for critical micelle concentration. Wang and Somasundaran [6] studied the electric double layer between CMC and talc using adsorption, electrophoretic mobility measurements, FTIR, fluorescence spectroscopy, AFM as well as molecular modeling. The obtained results showed that the adsorption of CMC on talc is affected by the pH and the ionic strength, which indicates the important role of electrostatic force in adsorption. Moreover, they concluded that the main forces responsible for CMC adsorption on talc are a combination of electrostatic interactions and hydrogen bonding. Morris et al. [2] measured the adsorption, electrokinetic and microflotation properties of CMC at the talc—water interface as the function of ionic strength and pH. According to their results, the amount of CMC adsorption increases with the decrease in pH and the increase in ionic strength, which was also confirmed by Pawlik et al. [7]. According to these authors, the increase in adsorption accompanies the increase in ionic strength, which results from the fact that the CMC macromolecules coil in solution to an extent that depends on the electrolyte concentration. Moreover, Shortridge et al. [8] studied the effect of chemical composition and molecular weight of CMC and modified guar gum reagents on the flotation of talc. They proved that guars were much more effective depressants of talc than the CMC samples when 0.001 mol dm^−3^ KNO_3_ was used as the background electrolyte. Parolis et al. [9] measured the effect of monovalent and divalent metal cations on the interactions between CMC and talc using adsorption, microflotation and intrinsic viscosity measurements. According to their results, calcium and magnesium ions increase the adsorption of CMC onto talc ions and promote the depression of talc by CMC. Another conclusion was that with the ionic strength less than 10^−1^, divalent cations caused greater coiling of CMC chains, but at an ionic strength higher than 10^−1^ the coiling effect was equivalent for divalent and monovalent cations. However, the adsorption of CMC on talc was still greater in the presence of divalent ions suggesting a specific interaction between the mineral surface, the divalent cations and CMC, which did not occur in the presence of K^+^ ions. Khraisheh et al. [10] conducted measurements on the influence of molecular weight and concentration on the adsorption of CMC onto talc at different ionic strengths. They found out that increasing the molecular weight of CMC results in an increase in CMC adsorption on talc. What is more, the addition of potassium, magnesium and calcium ions to the system increased the tendency of the polymer to adsorb onto the talc planes.

As can be seen, the papers analyzing the adsorption of CMC on the mineral surface concerning the influence of pH, ionic strength, type of used electrolyte and CMC molecular weight are frequent. Contrariwise, the influence of surfactants on the adsorption and the electrokinetic properties of the polysaccharide—metal oxide system has been neglected. The aim of this paper is to analyze the influence of surfactants, i.e., anionic sodium dodecylsulfate (abbreviated as SDS), non-ionic *tert*-octylphenol ethoxylate with an average of 9.5 ethylene oxide groups (abbreviated in what follows as TPO9) and their mixtures with the molecular ratios: 1:1; 1:3 and 3:1 on the adsorption of CMC on the MnO_2_ surface, as well as to analyze the electrokinetic properties of the carboxymethyl cellulose/manganese dioxide system in the presence of surfactants. All measurements were conducted in 0.001 mol dm^−3^ NaCl.

Carboxymethyl cellulose is an anionic polysaccharide obtained from cellulose, monochloroacetic acid and sodium hydroxide. Each CMC unit contains –CH_2_COO^−^ and –OH groups enabling formation of hydrogen bonds (Table 1). The molecular weight of this polymer ranges from 10^3^ to 10^6^. The maximal, theoretical value of its degree of substitution (DS), i.e., the average number of carboxymethyl groups per anhydroglucose unit, is 3, but for the commercial samples of CMC it is usually from 0.5 to 1.5 [10]. CMC is used in many branches of industry including: mineral processing, medicine, food, cosmetics, textiles and paints [6]. Manganese dioxide occurs in nature as a mineral pyrolusite. It is insoluble and stable over a broad pH range. This oxide finds application in the production of matches, the glass-making industry for decolorization of glass and as a depolarizer in voltaic cells [11].

**Table 1:**
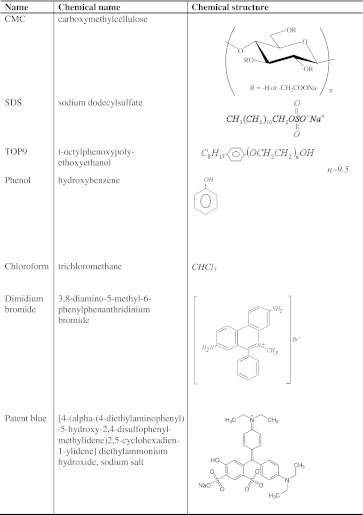
Names and structures of organic chemical compounds used in the measurements

## Experimental

### Materials

MnO_2_ produced by POCh Gliwice (Poland) was used as the adsorbent. The BET specific surface area for the sample was found to be 35 m^2^ g^−1^. The particle size distribution of MnO_2_ sample determined with the use of a Malvern Mastersizer 2000, fell entirely in the range from 1.82 to 22.71 μm, with a volume average size of 6.78 μm. The adsorbent was washed with double-distilled water until the conductivity of the supernatant was smaller than 2 μS cm^−1^. The XRD measurements confirmed that MnO_2_ was free of impurities.

The sodium salt of carboxymethylcellulose (CMC) of high viscosity was obtained from Sigma-Aldrich (the product number C5013). The viscosity average molecular weight measured using a rotary rheometer CVO 50 (Bohlin Instruments) was found to be 22,000. This value was calculated using the Mark-Houwink equation [12]. The degree of substitution was measured using the potentiometric titration. This value was determined at 1.02 ± 0.01. The exemplary formula of CMC is presented in Table 1. All CMC solution were prepared by slowly adding 0.25 g CMC powder into 250 ml of hot, vigorously stirred double-distilled water and further stirring for 30 min. The solution was refrigerated overnight to ensure complete hydration or dissolution.

Sodium dodecyl sulfate (SDS) and *tert*-octyl phenol ethoxylate with an average of 9.5 ethylene oxide groups per molecule, brand name Triton X-100 (abbreviated TOP9 in what follows), were purchased from Fluka. The concentrations of the surfactants and their mixtures in all the measured systems were 10^−4^ mol dm^−3^. Such a value prevents it from the exceeding the critical micelle concentration (c.m.c). It is very important because, after exceeding this value, molecules of surfactants form micelles. The interaction between these aggregates and polymer macromolecules are completely different from the interactions between monomers of surfactants and polysaccharide chains.

This is why it is very important to prevent surfactant molecules from forming micelles. There are many reports about critical micelle concentrations of SDS and TOP9 determined at different temperatures and by different methods. For SDS the critical micelle concentration is around 0.00825 mol dm^−3^ whereas for TOP9 the critical micelle concentration is around 0.00029 mol dm^−3^ [13–18]. 0.001 mol dm^−3^ NaCl produced by Fluka was used as the supporting electrolyte. The experiments were carried out in double-distilled water at room temperature (≅25 °C).

### Methods

#### Adsorption Measurements

A 0.2-g amount of manganese dioxide was added to 10 ml of solution prepared from the polymer stock solution (CMC), electrolyte (NaCl), doubly-distilled water and surfactants (SDS, TOP9) or their mixture with the molar ratios 1:1; 1:3 and 3:1). Next pH was adjusted to the desired value using 0.1 mol dm^−3^ HCl and 0.1 mol dm^−3^ NaOH. Six different initial concentrations of CMC were used (25–300 ppm). The suspension was shaken for 20 h, to achieve adsorption–desorption equilibrium, using a thermostated stirrer. To determine the CMC adsorption amount, the colorimetric method described by Dubois et al. [19] was used. For this, 0.05 ml 80 % phenol and 5 ml 98 % sulfuric acid were added to 2 ml of supernatant obtained after centrifugation with a speed of 14,000 rpm using a high speed centrifuge (310b Mechanika Precyzyjna). Time of centrifugation was 15 min. After 30 min of color development the absorbance was measured at a wavelength of 490 nm using a spectrophotometer (Specord M42, Carl Zeiss) with the computer program M500. All measurements were done in triplicate and the average values are reported. The amount of CMC adsorption on the MnO_2_ surface was calculated from a calibration curve according to the concentration difference before and after the adsorption tests.

The SDS concentration was analyzed by a variation of the Zerbe et al. method [20]. 1 dm^3^ of indicator solution was prepared by dissolving 0.16 g of dimidium bromide and 0.04 g of patent blue in double-distilled water in the presence of 40 ml of 1.25 M sulfuric acid. Next, in a separation funnel, 0.5 ml of sample solution was mixed with 39.5 ml pure water, followed by the addition of 10 ml of indicator solution and 20 ml of chloroform. The mixture obtained was vigorously shaken for 1 min and allowed to separate in to phases. The spectrophotometric measurement of chloroform solution at 526 nm was performed, using pure chloroform as a reference. The SDS concentration in the measured solutions was calculated from a calibration curve. All measurements were done in triplicate and the average values are reported. The measurement uncertainty in the analyses was from 4 to 8 %.

The TOP9 concentration was determined directly by UV absorbance at a wavelength 278 nm with doubly-distilled water as the Ref. [21]. All measurements were done in triplicate and the average values are reported. The measurement uncertainty in the analyses was from 1 to 3 %.

#### Potentiometric Titration

The surface charge on the metal oxide is formed as a result of reactions between the surface hydroxyl groups and electrolyte ions [22]. In aqueous solutions hydrogen/hydroxide ions as well as ions of background electrolyte are the most important in the surface charge formation process. Hydrogen ions influence the surface charge through the reactions of surface hydroxyl groups:

1 $$ \equiv SOH_{2}^{ + } \leftrightarrow \, \equiv SOH + H^{ + } $$

2 $$ \equiv SOH \leftrightarrow \, \equiv SO^{ - } + H^{ + } $$

In the classic theories of the electric double layer, background electrolyte ions are assumed to adsorb non-specifically, but in modern models, these ions also undergo specific adsorption. Ions become specifically adsorbed when short-range interactions between them and the interface become important. They are then believed to penetrate into the inner layer and may (but not necessarily) come into contact with the surface. They are usually assumed to form a partial or complete monolayer. On the other hand, ions are non-specifically adsorbed (positively or negatively) when they are subjected in the interphase only to long-range coulombic interactions (attraction or repulsion). They are believed to retain their solvation shell, and in the position of closest approach to the interface they are separated from it by one or more molecular layers.

A comparison between a titration curve of electrolyte and that of the metal oxide suspension of the same ionic strength is made for determination of the surface charge density of metal oxide. The surface charge density is calculated from the dependence between the volume of acid/base added to the suspension in order to obtain the desired pH value:

3 $$ \sigma_{0} = \frac{\Updelta VcF}{mS} $$

where: Δ*V*—the dependence between volume of acid/base added to the suspension in order to obtain the desired pH value, *c*—the molar concentration of acid/base, *F*—the Faraday constant (9.648 × 10^4^ C mol^−1^), *m*—the mass of metal oxide, *S*—the specific surface area of metal oxide.

The MnO_2_ surface charge density in the presence and absence of CMC and surfactant (SDS, TOP9 and their mixtures with the molecular ratios: 1:1; 1:3 and 3:1) was determined using the potentiometric titration method. The NaCl concentration was 0.001 mol dm^−3^. A thermostated Teflon vessel with a shaker, an automatic burette (Dosimat 665, Methrom) and a pH-meter were the parts of the measurement set. The process was controlled by a computer. The density of the MnO_2_ surface charge was determined using the “Miar_t” program written by W. Janusz. The surface charge density measurements were done in triplicate for every measured system. The results were obtained with a measurement uncertainty lower than 4 %.

#### Zeta Potential Measurements

A 0.05-g amount of manganese dioxide was added to 500 ml of the supporting electrolyte solution (0.001 mol dm^−3^ NaCl) with or without CMC and surfactants (SDS, TOP9 and their mixtures with the molecular ratios: 1:1; 1:3 and 3:1). The suspensions obtained were ultrasonicated for 10 min. Then the pH was adjusted and the electrophoretic mobility was measured using a zetameter (Zetasizer 3,000, Malvern Instruments) and then the zeta potential *ζ* was calculated from the Smoluchowski equation [23]. The zeta potential measurements were done in triplicate and the results were obtained with a measurement uncertainty from 3 to 7 %. In the paper the average values are reported.

#### Thickness of the Polymer Adsorption Layer

The thickness of the polysaccharide adsorption layer (*δ*) was determined from the viscosity measurements [24], using a rheometer (CVO 50, Bohlin Instruments). Polysaccharide adsorption on the solid surface causes an increase in the solid particle radius which gives the adsorption layer thickness (*δ*). It results in an increase in the volume fraction (*ϕ*_*0*_) of the dispersed solid. Thus the *δ* values were obtained from the dependence:

4 $$ \delta = r\left[\left( {\frac{{\phi_{\text{p}} }}{{\phi_{ 0} }}} \right)^{1/3} - 1\right] $$

where: *r*—the radius of the metal oxide particle, *ϕ*_p_—the volumetric fraction in the presence of polymer, *ϕ*_0_—the volumetric fraction in the absence of the polymer.

The Einstein equation connects the volume fraction of the dispersed solid with the suspension viscosity in the following way:

5 $$ \frac{\eta }{{\eta_{0} }} = 1 + k\phi_{0} $$

where: *η* is the viscosity of the suspension (Pa s), *η*_0_ is the viscosity of the liquid phase (Pa s), and *k* is the Einstein coefficient. The coefficient *k* is equal to 2.5 for the rigid spherical particles in infinitely diluted suspensions.

The volumetric fraction (*ϕ*_p_) in the presence of a polymer or a polymer-surfactant complex was determined from the linear dependency of *η*/*η*_0_ versus *ϕ*_0_ of manganese dioxide (calibration curve). The viscosity measurements enabling the *η*/*η*_0_ ratio determination in the presence of a polymer or a polymer-surfactant complex were made with the volume fraction of MnO_2_ equal to 13.7 × 10^−3^. Because the adsorption of a polymer or a polymer surfactant complex caused an increase in the ratio, the value *ϕ*_p_ was determined directly from the calibration curve (as a magnitude related to this ratio). Then the thickness of the polysaccharide adsorption layer was calculated (Eq. 4).

## Results and Discussion

**Fig. 1:**
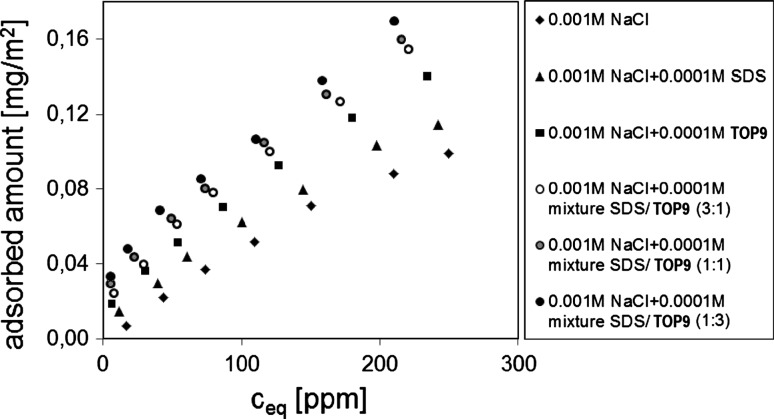
Adsorption isotherms of CMC on MnO_2_ in the presence and absence of surfactants in 0.001 M NaCl, pH = 6

Figure 1 presents the Langmuir adsorption isotherms of CMC on the MnO_2_ surface in the presence or absence of the surfactant (SDS, TOP9 and their mixtures SDS/TOP9 with the molar ratios: 1:1; 1:3; 3:1). Measurements were performed in 0.001 mol dm^−3^ NaCl as a background electrolyte. As mentioned in the Introduction, despite many measurements of polysaccharides adsorption [25–28], the mechanism of this process is far from being understood. It is known that CMC adsorption results from hydrophobic and (or) electrostatic interactions. The carboxylic and hydroxyl groups from CMC can interact with the metallic species from the mineral surface [29]. However, the carboxyl groups can interact with various forms of metallic ionic species whereas the hydroxyl groups can interact mostly with the metal hydroxyl species [5]. The reaction between the polymer macromolecules and the metal hydroxyl groups from the solid surface together with hydrogen bonding and hydrophobic interaction are the most typical adsorption mechanisms [1–4]. The above mentioned mechanisms change dramatically when the third substance such as a surfactant is added to the measured system. The reasons for this are interactions between the surface active agents and polysaccharides. There is much evidence in the world literature that surfactants are likely to form complexes with polysaccharides. Terada et al. [30] measured the influence of SDS on the adsorption of hydroxyethyl cellulose and hydrophobically modified cationic cellulose. They found out that complexes are formed between the above-mentioned polysaccharides and SDS molecules. This was also confirmed by Samoshina et al. [31]. Moreover, Liu and et al. [32] proved the existence of complexes between sodium carboxymethylcellulose and C12mimBr (1-dodecyl-3-methylimidazolinum bromide) using the isothermal titration microcalorimetry, turbidimetric titration and surface tension measurements. As it may be deduced from Fig. 1, the adsorption of carboxymethylcellulose increases in the presence of surface active agents or their mixtures. This increase is the smallest in the presence of anionic SDS, larger in the presence of non-ionic TOP9, and even larger in the presence of surfactant mixtures SDS/TOP9. Among these mixtures the largest increase in the amount of adsorption of CMC on the MnO_2_ surface is observed in the presence of surfactant mixtures with the molar ratio 1:3. This observed increase in CMC adsorption in the presence of surfactants is a consequence of the formation of complexes between the polysaccharide macromolecules and the surfactants molecules. Unfortunately, the nature of these complexes has not been fully understood. When SDS is added to the adsorption system, the complexes formed are definitely non-electrostatic because CMC and SDS are of the same charge. Possible mechanisms of the formation of these complexes are hydrophobic interactions and hydrogen bonds between the CMC and SDS molecules. However, the possibility of complex formation between CMC and SDS in the presence of SDS is smaller than in the presence of non-ionic TOP9. The reason for that is the electrostatic repulsion between the CMC macromolecules and SDS. In the presence of TOP9 the increase in CMC adsorption is higher. This surfactant does not form any charge and has a great capability to form complexes [33]. Figure 1 shows also that among measured surfactant mixtures, the adsorption of CMC is the lowest in the presence of the mixture with the molar ratio 3:1, a bit higher in the presence of the mixture 1:1 and the highest when a SDS/TOP9 mixture with the molar ratio 1:3 is used. All these mixtures exhibit a strong synergetic effect [34, 35], resulting in an increase in adsorptive, foaming and rewetting properties of the surfactant mixtures in comparison to pure surfactant solutions. This effect is clearly visible in the analyzed system. Moreover, the obtained data proved also that TOP9 interacts with the CMC macromolecules more than SDS, which is in agreement with the results obtained in the presence of single surfactant solutions.

**Fig. 2:**
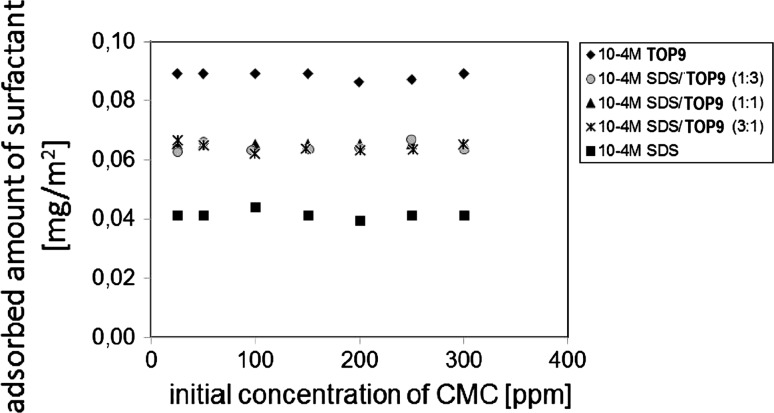
Influence of the initial concentration of CMC on the adsorbed amount of surfactants 0.001 M NaCl, pH = 6

Figure 2 presents the influence of the initial concentration of CMC on the adsorbed amount of surfactants. Measurements were performed at pH ≅ 6 and in the presence of 0.001 mol dm^−3^ NaCl. These results help prepare a comprehensive analysis of the examined adsorption systems. The analysis of the presented data shows that surfactants adsorption does not depend on the CMC initial concentration and it is constant in the measured concentration range. What is more, all surfactants added to the adsorption systems as well as their mixtures are nearly entirely adsorbed on the MnO_2_ surface. Unfortunately is impossible from the adsorption data to estimate if surfactants adsorbed directly on the surface of the metal oxide or as complexes with CMC. However, electrokinetic measurements allow to give the answer to that question.

**Fig. 3:**
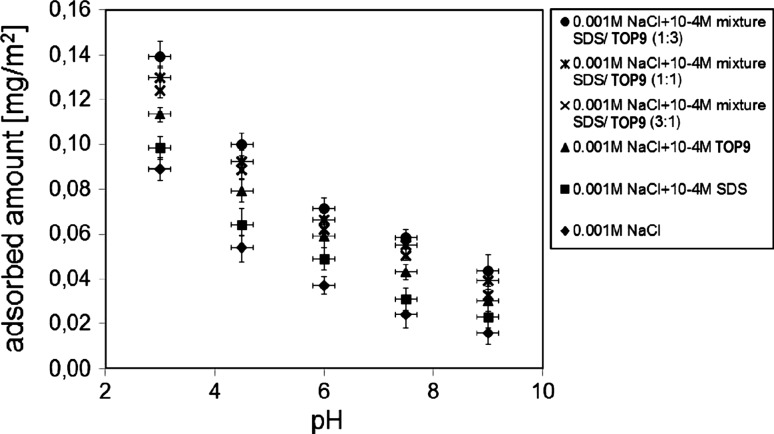
Influence of pH on the adsorption of CMC (200 ppm) on MnO_2_ in the presence of 0.001 M NaCl

Figure 3 shows the influence of pH on the CMC adsorption amount on the MnO_2_ surface. As one can see, the increase in pH accompanies the decrease in CMC adsorption in every measured adsorption system. This situation is the consequence of the influence of pH on the dissociation degree of CMC and the kind and concentration of the surface active MnO_2_ groups. It is known that manganese dioxide up to pH 4–5 is positively charged (pH_pzc_ = 4–5 [36]) and the CMC chains contain a lot of nondissociated groups, the most likely adsorption mechanism is the formation of hydrogen bonds. A further increase in pH causes an increase in a number of negatively charged surface active groups on the metal oxide surface. As CMC, as the anionic polyelectrolyte, also has a negative value, the decrease in CMC adsorption on MnO_2_ surface with the increase in pH results from the electrostatic repulsion between the surface of the adsorbent and the polymer chains. Moreover, the adsorption of CMC on MnO_2_ with high pH values evidences the specific interaction between the chains of CMC and the surface groups of manganese dioxide. Another important factor which also has an influence on the decrease in CMC adsorption with the increase in pH is a conformation of the adsorbed polymer chains. The increase in pH causes an increase in a number of negatively charged groups. If the groups belong to the same chain they can repel each other. Because of this repulsion the polymer chain tends to become straight. The adsorption of such a straight chain impedes the access to the rest of the active centers of the metal oxide. In such a situation the amount of the adsorbed polymer dramatically decreases.

**Fig. 4:**
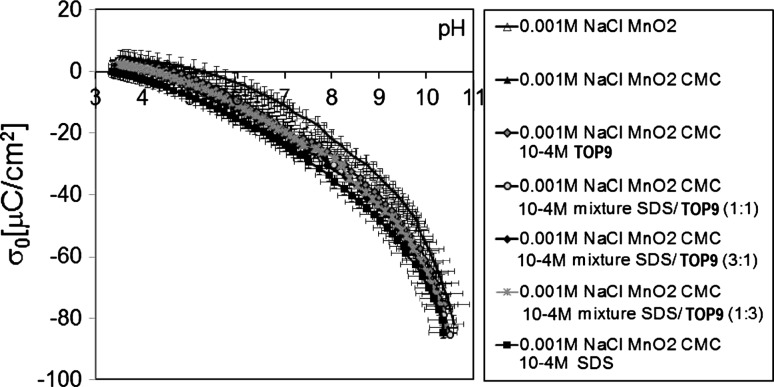
Surface charge density in the system: MnO_2_/0.001 M NaCl in the presence of 100 ppm CMC and 10^−4^ M surfactants

In order to prepare a comprehensive analysis of the metal oxide/polysaccharide/surfactant/electrolyte system the surface charge density and zeta potential measurements were conducted. Figure 4 shows the influence of pH, CMC, surfactants (SDS, TOP9) and their mixtures (SDS/TOP9 with the molar ratio 1:3; 1:1; 3:1) on the surface charge of manganese dioxide. As one can see, the surface charge of MnO_2_ strongly depends on the pH of the solution. The point of zero charge for this dioxide is at pH 4–5. At a pH lower than 4, the metal oxide is positively charged because of a high concentration of MnOH_2_^+^ groups. At pH values higher than the pH_pzc_, the oxide surface is negatively charged because of a high concentration of MnO^−^ groups. Another observation is that the presence of anionic CMC and all measured surfactants causes a decrease in the surface charge of MnO_2_ in the whole measured pH range as well as a shift of the point of zero charge towards lower pH. This phenomenon results from the presence of the negatively charged groups from the CMC and SDS molecules or from the polysaccharide/surfactant complexes: CMC/SDS, CMC/TOP9, CMC/SDS/TOP9. These negatively charged groups are not linked with the surface but present in the compact part of the electric double layer [37]. Moreover, there are no significant differences between the surface charge density of MnO_2_ in the presence of different surfactants. As one can see, the lowest values of surface charge density were observed in the presence of CMC and SDS. In this particular adsorption system the number of negatively charged carboxylic groups in the compact part of the electric double layer is the highest which causes the decrease in MnO_2_ surface charge density. However, the observed differences between the measured adsorption systems are very small. This observation is evidence that the surfactants are not directly linked with the metal oxide surface but they are adsorbed onto the surface as complexes with CMC. This is why there are differences in the zeta potential values of MnO_2_ in the presence of surfactants.

**Fig. 5:**
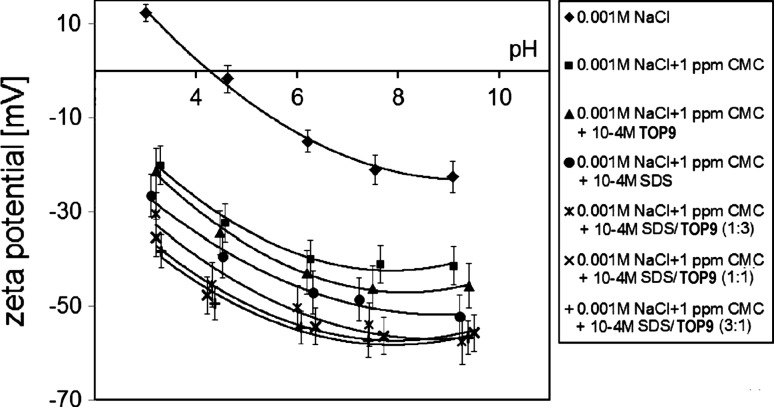
Influence of CMC and surfactants (SDS, TOP9 and their mixtures SDS/TOP9 with the molar ratios: 1:1; 1:3; 3:1) on the zeta potential of MnO_2_ in the presence of 0.001 M NaCl

Figure 5 presents the influence of pH, CMC, surfactants (SDS, TOP9) and their mixtures (SDS/TOP9 with the molar ratios: 1:1; 1:3; 3:1) on the zeta potential of manganese dioxide. It is clearly visible that the presence of CMC and surfactants also causes a decrease in the zeta potential and a shift of the isoelectric point (pH_iep_) of MnO_2_ towards a lower pH (pH_iep_ for MnO_2_ ≅ 4). As can be seen, the zeta potential of MnO_2_ is the highest in the pure electrolyte solution a bit lower in the presence of CMC, then in the adsorption system connected with CMC and TOP9, much lower in the presence of the CMC/SDS mixture and the lowest in the presence of CMC and surfactant mixtures (SDS/TOP9). Of the measured surfactant mixtures, the zeta potentials do not differ a lot. There are two factors responsible for the decrease in the zeta potential of the metal oxide. The first one is the presence of negatively charged groups from CMC or SDS molecules in the diffused part of the electric double layer [38], the second is the shift of the slipping plane towards the bulk solution. This shift results from the adsorption of macromolecules of polysaccharide or the polysaccharide/surfactant complexes. The smallest decrease in the zeta potential is observed in the pure electrolyte solution. The reason for this is the lack of negatively charged groups in the diffused part of the electric double layer and also the lack of the shift of the slipping plane. In the CMC/MnO_2_ system decrease in the zeta potential results from the presence of negatively charged carboxylic groups from the CMC macromolecules as well as from the shift of the slipping plane. A bit larger decrease in the zeta potential in the presence of CMC and TOP9 is a consequence of complexes formation between the surfactant molecules and the polysaccharide macromolecules as well as the negative charge from the CMC macromolecules. Larger decrease in the zeta potential occurs in the presence of CMC and SDS. In this adsorption system concentration of negatively charged groups is the highest. These groups together with the shift of the slipping plane are responsible for the decrease in the zeta potential of MnO_2_. The largest decrease in the zeta potential of manganese dioxide is observed in the presence of CMC and the surfactant mixtures SDS/TOP9. It means that in such systems, the adsorption layer is the most expanded towards the bulk solution. These mixtures exhibit a strong synergetic effect [39]. Among them the values of the zeta potential obtained are quite similar. These data are in agreement with the results of polysaccharide adsorption layer thickness.

Table 2 presents the thickness of the CMC adsorption layer on the surface of manganese dioxide in the presence of surfactants and their mixtures. The data obtained let us draw some conclusions about the structure of the adsorbed polymer layers.

**Table 2:** The influence of surfactants on the thickness of polysaccharide adsorption layer in the system: CMC/MnO_2_; c_CMC_ = 100 ppm; c_NaCl_ = 0.001 mol/dm^3^; c_surf_ = 0.0001 mol/dm^3^; pH = 6

System	δ (nm)
CMC/NaCl/MnO_2_	4.6
CMC/NaCl/TOP9/MnO_2_	6.2
CMC/NaCl/SDS/MnO_2_	10.1
CMC/NaCl/(SDS + TOP9 (1:3))/MnO_2_	13.5
CMC/NaCl/(SDS + TOP9 (1:1))/MnO_2_	14.0
CMC/NaCl/(SDS + TOP9 (3:1))/MnO_2_	14.3

The increase in CMC adsorption layer thickness on the MnO_2_ surface in the presence of surfactants and their mixtures is observed. This fact is a consequence of a few phenomena. Firstly, CMC and SDS have negatively charged groups and because of their repulsion, the polysaccharide forms a conformation expanded towards the bulk of solution which also leads to an increase in the CMC adsorption layer thickness. Secondly, all of these surfactants may form complexes with CMC macromolecules. The adsorption of CMC/surfactant complexes on the metal oxide surface causes an increase in the adsorption layer thicknesses. This increase is a consequence of a conformation rich in spacious structures.

## Conclusions

The results obtained prove that the adsorption of CMC and complexes between CMC and surfactants strongly influences the structure of the electric double layer MnO_2_/electrolyte. First of all the presence of surfactants (SDS, TOP9) as well as their mixtures (SDS/TOP9 with the molar ratio 1:3; 1:1 and 3:1) causes the increase in CMC adsorption amount on the MnO_2_ surface in every measured system. This phenomenon is a consequence of the formation of complexes between the CMC macromolecules and the surfactant molecules. These complexes are created more effectively with non-ionic TOP9 and anionic CMC than with negatively charged SDS and CMC. Secondly, the adsorption of CMC macromolecules or complexes between CMC and surfactants causes a decrease in the surface charge of MnO_2_ and the shift of the point of zero charge to a lower pH. The main reason for that is the presence of the negatively charged groups from CMC or CMC and anionic SDS. Thirdly, the presence of CMC and surfactants also causes a decrease in the zeta potential and the shift of the isoelectric point (pH_iep_) of MnO_2_ towards a lower pH. Such a phenomenon results from the shift of the slipping plane towards the bulk solution, as well as from the presence of negatively charged groups in the diffused part of the electric double layer. The last conclusion is that the addition of surfactants to the adsorption system also has an influence on the structure of the polysaccharide adsorption layer on the MnO_2_ surface. In the presence of surfactants, the adsorption layer is expanded towards the bulk solution and the order of the data obtained is in agreement with that of the MnO_2_ zeta potential values.
